# High-dose fish oil supplements are more effective than oily fish in altering the number and function of extracellular vesicles in healthy human subjects: a randomised, double-blind, placebo-controlled, parallel trial

**DOI:** 10.1017/S0007114525000625

**Published:** 2025-04-14

**Authors:** Amal Sharman, Ruihan Zhou, Jamie Pugh, Graeme Close, Helena L. Fisk, Philip C. Calder, Parveen Yaqoob

**Affiliations:** 1 Hugh Sinclair Unit of Human Nutrition, Department of Food and Nutritional Sciences, School of Chemistry, Food & Pharmacy, University of Reading, Reading, UK; 2 Schools of Sport and Exercise Science, Liverpool John Moores University, Liverpool, UK; 3 School of Human Development and Health, Faculty of Medicine, University of Southampton, Southampton, UK and NIHR Southampton Biomedical Research Centre, University Hospital Southampton NHS Foundation Trust and University of Southampton, Southampton, UK

**Keywords:** CVD, Extracellular vesicles, Fish oil, Oily fish, Thrombosis, Coagulation

## Abstract

*n*-3 PUFA delivered by fish oil supplements alter the number and functions of circulating extracellular vesicles (EV), but consumption of oily fish does not reproduce this effect. In order to assess the effects of fish oil supplements and oily fish, at a level achievable in the diet, on EV numbers, composition and procoagulant activity in healthy human volunteers, forty-two healthy subjects were assigned to one of three treatment groups: (i) fish oil supplements plus white fish meals, (ii) control supplements plus oily fish meals or (iii) control supplements plus white fish meals for 12 weeks in a randomised, double-blind, placebo-controlled, parallel trial; circulating EV were enumerated and their procoagulant activity assessed using thrombin generation and fibrinolysis assays. Our results showed that fish oil supplements decreased circulating EV numbers and reduced EV-stimulated thrombin generation, but the consumption of oily fish at half the dose of EPA had no effect on either EV number or thrombogenic capacity. Consumption of both oily fish and fish oil supplements increased the EPA and DHA contents of EV, and the proportion of EPA in circulating EV was strongly associated with EV-stimulated thrombin generation. This study revealed that the additional 1 g/d EPA delivered in the fish oil supplements is required to decrease the numbers and thrombogenic capacity of EV, since oily fish at a level achievable in the diet had no effect. Increasing EPA intake beyond current guidelines for oily fish consumption may therefore be required for cardiovascular benefits relating to EV.

Extracellular vesicles (EV) are lipid bilayer-enclosed vesicles naturally present in the bodily fluids of healthy individuals and derived from almost all cells under both physiological and pathological conditions. The properties and functions of EV are diverse and are primarily determined by their cellular origin, the stimulus triggering their release and their specific cargo, which includes nucleic acids, proteins and lipids^(1)^. EV contribute to the regulation of normal physiological processes, such as blood coagulation, intercellular communication and tissue repair^(2,3)^ and may serve as potential biomarkers for diseases, such as CVD, type 2 diabetes and cancer^(4–7)^. There is particular interest in the association of EV numbers with endothelial dysfunction^(7)^, platelet activation^(8)^, vascular inflammation^(9)^ and the aspects of blood coagulation pathways^(10)^, as well as being correlated with CVD and metabolic syndrome^(4,7,11)^, all of which suggest that EV have potential as a novel biomarker of cardiometabolic diseases.

Dietary *n*-3 PUFA, which are abundant in oily fish and fish oils, have long been associated with protection from CVD and although the strength of evidence has at times been questioned, the most recent meta-analyses and systematic reviews broadly support the view that *n*-3 PUFA supplementation lowers the risk of either CVD-related death or all-cause mortality or both^(12,13)^. Furthermore, a recent analysis of seventeen prospective studies demonstrated that the risk of death from all causes and death from CVD was significantly lower in the highest *v*. the lowest quintile for circulating *n*-3 PUFA^(14)^. A range of potential mechanisms of this cardiovascular benefit have been considered^(15)^, but to date, very few studies have explored the effects of *n*-3 PUFA on EV numbers and/or function. A small number of studies have demonstrated a decrease in the number of EV after the intervention with *n*-3 PUFA supplements^(16–20)^. We have previously demonstrated that supplementation with *n*-3 PUFA decreased the numbers of circulating total EV, EV subtypes from platelets and endothelial cells, as well as their coagulatory behaviour, in individuals with moderate risk of CVD^(21)^. However, there is no information about whether oily fish, consumed at a level that is achievable through the diet, affects either EV numbers or function. Furthermore, there is a lack of insight as to whether EPA and DHA perform differently in relation to effects on EV. The current study therefore examines, for the first time, whether *n*-3 PUFA delivered in the form of oily fish is able to modify the profile and coagulatory behaviour of EV in the circulation in the same way as fish oil supplements, consumed at a dose sufficient to lower numbers of EV, and whether the effect is driven by EPA or DHA.

## Methods

This study was conducted at the School of Sport and Exercise Sciences, Liverpool John Moores University, from October 2016 January 2017 and carried out according to the guidelines in the Declaration of Helsinki, with ethical approval from the National Research Ethics Service (S16SPS041). Written informed consent was obtained from participants.

### Trial design

The trial was a randomised, double-blind, placebo-controlled, parallel trial. Eligible participants were allocated randomly (block randomisation was performed using Excel by a member of staff unrelated to the trial) to one of the three groups as follows: (i) fish oil capsules provided as 2·2 g/d of *n*-3 PUFA ethyl esters plus two white fish meals per week, (ii) control capsules containing refined olive oil plus two oily fish meals providing the equivalent of 2·2 g/d of *n*-3 PUFA (one meal containing salmon and the other mackerel) and (iii) control capsules plus two white fish meals (Table 1). Although the doses of EPA were not matched, they represented optimal levels of intake which could be achieved through diet or supplementation, and in the case of the supplements, a dose which previous evidence has shown would be sufficient to lower the number of EV^(20)^. The fish meals were provided as ready meals supplied by Soulmate Food (Liverpool, UK), and the capsules were supplied by Wiley’s Finest (Granville, Ohio, USA). Servings of mackerel, salmon and white fish contained within the ready meals were 229 g, 240 g and 110 g respectively. The fatty acid compositions of the fish oil capsules, control capsules and fish meals were analysed by the West Yorkshire analytical services (Leeds, UK) and are shown in Table 1. Capsules were coded by an independent researcher, and the code was broken after all data analysis had been completed. All meals were delivered chilled and distributed to participants twice a week, with storage and heating instructions provided by the supplier. Participants attended three visits during the intervention: at screening, baseline and at the end of the intervention (12 weeks). Compliance with the intervention was confirmed verbally during each week of the trial. A 3-day food diary was administered 3 days prior to the start of the intervention and during the last 3 days of the intervention to confirm that participants maintained consistent habitual dietary intakes during the study. Researchers involved in the measurement and assessment of study outcomes generated the random allocation sequence, enrolled the participants and assigned the interventions, but were blinded to the allocation of treatment; all participants were blinded to the interventions as well.

**Table 1.** Fatty acid compositions of fish oil, control oil capsules and whole homogenised fish meals

	Fish oil capsules		Control capsules (olive oil)			Piri piri fish (Salmon)	Katsu fish curry (Mackerel)	Piri piri fish (White fish)
Fatty acids	g/capsule	(wt%)	g/capsule	(wt%)	Parameters	g/portion	g/portion	g/portion
Myristic acid (14:0)	0·0003	0·0375	0·0001	0·0198	Meal weight	534	586	378
Tetracosanoic (24:0)	0·0038	0·3875	0·0007	0·0761	Fish weight	226	229	110
Oleic acid (18:1*n*-9)	0·0042	0·425	0·6694	66·945	Total calories	837 kcal	845 kcal	861 kcal
Linoleic acid (18:2*n*-6)	0·0016	0·1625	0·0871	8·7133	Protein	69	70	72
DGLA (20:3*n*-6)	0·0018	0·1875	0	0	Carbohydrates	56	57	58
AA (20:4*n*-6)	0·0195	1·95	0	0	Fat	36	36	37
ALA (18:3*n*-3)	0·002	0·2	0·0065	0·6567	ALA (18:3*n*-3)	0·7	0·9	0·6
ETA (20:4*n*-3)	0·0206	2·0625	0	0	SA (18:4*n*-3)	1·6	4·5	0·2
EPA (20:5*n*-3)	0·7543	75·437	0·0003	0·03208	EPA (20:5*n*-3)	0·7	2·2	0·04
HPA (21:5*n*-3)	0·0197	1·975	0	0	DPA (22:5*n*-3)	0·3	0·4	<0·04
DPA (22:5*n*-3)	0·0166	1·6625	0·0005	0·0506	DHA (22:6*n*-3)	0·7	3·2	0·1
DHA (22:6*n*-3)	0·2671	26·712	0·0006	0·0604	Total *n*-3 PUFA	4·0	11·2	0·94
Total SFA	0·0042	0·425	0·1401	14·011				
Total MUFA	0·0108	1·0875	0·6771	67·718				
Total *n*-3 PUFA	1·1038	110·38	0·0079	0·7998				
	g/d (2 capsules)					g/d (oily fish)		
Total *n*-3 PUFA	2·2				Total *n*-3 PUFA	2·2		
EPA	1·5				EPA	0·4		
DHA	0·53				DHA	0·56		
EPA + DHA	2				EPA + DHA	1		

Data are expressed as grams per capsule (g/capsule) and the percentages of the weight of each individual fatty acid relative to the total weight of all fatty acids (wt%) in either fish oil or control oil capsules, and grams per portion (g/portion) in fish meals. AA, arachidonic acid; ALA, *α*-linolenic acid; DGLA, dihomo-γ-linolenic acid; DPA, docosapentaenoic acid; ETA, eicosatetraenoic acid; GLA, γ-linolenic acid; HPA, heneicosapentaenoic; SA, stearidonic acid.

### Participants

Participants older than 40 years were recruited through the media (BBC local and national radio, online forums, social media, societies and flyers in the street) and a total of forty-two participants completed the study (54·97 ± 1·45 years; 28 females), as illustrated in the participant flow diagram (online Supplementary Figure 1). The Framingham Risk Score was used to identify participants who were at above average risk of developing CVD^(22)^, defined as a relative risk of 1·5 based on scoring a minimum of two points against one or more of the criteria listed in online Supplementary Table 1, which includes family history of myocardial infarction or type 2 diabetes^(23)^. Exclusion criteria included the following: smoking; infection; fever of unknown origin; immune disorders, including HIV; autoimmune diseases; medical conditions requiring immediate intervention; unstable or rapidly progressive neurological diseases; a history of hemorrhagic or ischaemic stroke within the last 3 months; consuming oily fish more than once per week on average and/or *n*-3 index > 6 %; taking any medication and/or dietary supplements; allergy, hypersensitivity or intolerance to fish, fish oils or *n*-3 fats; any known food allergies; alcohol misuse; pregnant or breastfeeding.

### Blood collection and processing

Venous blood samples were collected into 3·2 % sodium citrate and processed to platelet-free plasma (PFP), as previously described^(24)^. PFP was aliquoted and stored at −80 °C for further analysis.

### Extracellular vesicle isolation

EV were isolated using size exclusion chromatography (SEC) (Izon, Oxford, UK). PFP (0·5 ml) was thawed at room temperature on a sample roller and loaded onto a qEV original column which had been pre-flushed with 30 ml PBS. A further 5 ml PBS was passed through the column to elute the EV based on their size and nine 0·5 ml fractions were collected. Fractions 7–9 were pooled for EV quantification and analysis.

### Enumeration and characterisation of extracellular vesicle

#### Nanoparticle tracking analysis

The concentration and size distribution of circulating EV was determined by nanoparticle tracking analysis using a NanoSight 300 (NS300; Malvern, Amesbury, UK)^(24)^. The size of circulating EV detected by nanoparticle tracking analysis ranged from 70 nm to approximately 350 nm, but the majority (> 80 %) were 100–200 nm.

#### Flow cytometry

The concentrations of circulating EV subpopulations including phosphatidylserine (PS) positive EV, platelet-derived EV (PDEV) and endothelial-derived EV were determined by flow cytometry (Canto II Flow Cytometer, BD Biosciences, UK), using blue (488 nm), a red (633 nm) and violet (405 nm) lasers^(24)^.

### Fatty acid compositions of RBC and extracellular vesicle

Lipid extracts were prepared from RBC and EV (isolated by SEC, as above) and separated by solid phase extraction. Briefly, pooled fractions of EV (800 µl) and RBC (50 µl) were mixed with 5 ml of chloroform/methanol (2/1) containing 50 mg/l butylated hydroxytoluene as an antioxidant and then centrifuged at 1000 × g for 10 min. The lower phase was collected and dried under nitrogen at 40 °C. Dry toluene (0·5 ml) was added to the total lipid extract, followed by methanol (1 ml) containing 2 % (v/v) sulphuric acid. The tubes were capped and incubated at 50 °C for 2 h. After cooling, samples were neutralised with 0·25M KHCO_3_/0·5M K_2_CO_3_ (1 ml), and lipid was extracted by adding dry hexane (1 ml) and centrifuged at 250 × g for 2 min at room temperature. The upper phase containing the fatty acid methyl esters was collected and analysed using gas chromatography on a Hewlett-Packard 6890 gas chromatograph (Hewlett-Packard, California, USA) equipped with flame ionisation detection (Agilent Technologies, Cheadle, UK). Fatty acid methyl ester was separated in a BPX-70 fused silica capillary column (30 m × 0·25 mm × 25 µm; SGE Analytical Science, UK) at a split ratio of 30:1 and an injection volume of 5 µl. The temperature of both injector and detector was kept at 300 °C, and the programme was set at an initial temperature of 115 °C for 2 min, increased at 10 °C/min to 200 °C, held at this temperature for 16 min and then finally increased at 60 °C/min to 240 °C for 2 min (total run time 29·2 min). Helium was used as carrier gas (velocity: 29 cm/s; pressure: 21:96psi and flow rate: 1·0 ml/min) and make-up gas, flow rate: 45 ml/min. Hydrogen was used as detector gas were a hydrogen flow rate of 40 ml/min and air flow of 120 ml/min. Samples were analysed by using ChemStation software (Agilent Technologies, Cheadle, UK) and Microsoft Excel (Microsoft Corporation, USA).

### Coagulatory function of extracellular vesicle

#### Measurement of thrombin generation

Thrombin formation was assessed using the Technothrombin MP kit (Technoclone, Vienna), which is based on the thrombin-dependent cleavage of a fluorogenic substrate over time, as previously described^(21)^. Two separate analyses were conducted: (i) determination of the effects of the intervention on thrombin generation in PFP from study samples compared with that in pooled vesicle-depleted plasma (VDP) alone and (ii) determination of the effects of the intervention on thrombin generation in VDP plus EV isolated by SEC from study samples compared with that in VFP alone. The methods for both approaches were based on the use of pooled VDP as a negative control to allow assessment of thrombin generation specifically resulting from the presence of EV, and VDP was prepared from three healthy volunteers as previously described^(21)^. For the first approach, 40 µl aliquots of either pre-thawed study sample PFP or pooled VDP were added to the plate. For the second approach, EV fractions were first eluted by SEC and were concentrated using Vivaspin™ 6 Sample Concentrators with 100 000 MWCO (Fisher Scientific, Loughborough) at 1500 × g for 40 min and EV aliquot (10 µl) with a final protein concentration of 5 µg/ml was then added to 30 µl of VDP. Data were then analysed by the TGA Evaluation Software manually to convert the unit of thrombin generation from RFU to nM and presented as lag time, peak concentration of thrombin (nM), velocity–index and area under the curve (AUC).

#### Clot formation and fibrinolysis

The clot-forming capacity of EV was assessed by isolating EVs using SEC, adjusting the concentration to 5 µg/ml and applying clot formation and lysis assays adapted from previous studies^(25,26)^ to compare clot formation and fibrinolytic activity in VDP with VDP plus added EV. The clot formation assay was performed in duplicate in 96 well plates by incubating EV (10 µl, final concentration 5 µg/ml) and VFP (30 µl) with Tween Tris-buffered saline (40 µl, containing 10 mM Tris pH 7·4; 0·01 % Tween 20 (T/T)) and 20 µl of 5·3 mM CaCl_2_. The clot was measured at 405 nm every 30 s for 1 h at 37 °C using a FlexStation 3 microplate reader (Molecular Devices, San Jose, USA). The fibrinolytic activity of EV was assessed by isolating EV and measuring their ability to initiate plasmin generation (enzyme important for degrading the blood clot) using a chromogenic assay. In brief, EV (10 µl, final concentration 5 µg/ml) were incubated with VFP (30 µl), Tween Tris-buffered saline (30 µl, containing 10 mM Tris pH 7·4; 0·01 % Tween 20 (T/T)), tissue plasminogen activator to stimulate clot breakdown (10 µl, final concentration 100 pM) and 20 µl of 5·3 mM CaCl_2_. The kinetics measurement was started immediately after adding the calcium, and readings were taken every 30 s at 405 nm for 4 h using a FlexStation 3 microplate reader (Molecular Devices, San Jose, USA). All data were analysed using an online tool for analysis of clot and lysis using the Shiny App developed by Longstaff^(27)^ and presented as AUC and time to full lysis, respectively.

### Plasma lipid analysis

The plasma lipid profile before and after the treatment was assessed using a Daytona Plus clinical chemistry analyser (Randox). Plasma total cholesterol and triacylglycerol were analysed using enzyme-based assays and HDL-cholesterol was analysed using a clearance assay, while LDL-cholesterol was estimated using the Friedewald formula.

### Power calculation

The sample size calculation was performed according to Julious *et al.* ^(28)^ for the primary endpoints of the proportions of both EPA and DHA in RBC, in which ten subjects would provide 80 % power in detecting a difference of 0·9 wt% in EPA of RBC with *α* = 0·05 and a within-subject standard deviation of 1·0, and fifteen subjects would provide 80 % power in detecting a difference of 1·6 wt% in DHA of RBC with *α* = 0·05 and a within-subject standard deviation of 0·8.

### Statistical analysis

Data are expressed as mean (standard error of the mean (sem)). Differences between three groups were determined using a general linear model with fixed factors of treatment and period followed by post hoc analysis using Bonferroni tests where applicable. The strengths of correlations among EV numbers and EV coagulatory functions with fatty acid compositions of both EV and RBC were assessed by Pearson’s correlation coefficient or Spearman’s correlation coefficient where appropriate. Significantly associated variables were entered into a multivariate regression model, and all variables with *P*-values < 0·05 were subsequently incorporated into a stepwise multivariate regression model, in which parameters of *F* ≤ 0·05 were entered and *F* ≥ 0·10 were removed, to identify independent predictors of EV-related parameters. *P*-values < 0·05 were considered statistically significant. Statistical analyses were performed using IBM SPSS statistics 27.

## Results

A total of forty-two participants aged 55 ± 2 years completed the 12-week intervention. The three groups did not differ significantly with regard to their physical characteristics, BP, lipid and glucose profile (Table 2).

**Table 2.** Baseline subject characteristics (Mean values with their standard errors of the mean)

	Fish oil supplement (*n* 15)		Oily fish (*n* 14)		Control (*n* 13)		*P* value
	Mean	sem	Mean	sem	Mean	sem	
Subject characteristics							
Age (years)	57·23	2·76	56·14	2·50	51·16	2·00	0·209
Male:female ratio	3:10^†^		5:9		2:9^†^		0·603
Height (m)	1·69	0·02	1·65	0·02	1·68	0·02	0·561
Weight (kg)	75·86	3·40	68·85	2·83	73·63	4·64	0·370
BMI (kg/m^2^)	27·10	1·45	25·16	0·98	25·83	1·50	0·675
SBP (mm Hg)	127·91	4·67	128·15	3·96	125·18	6·13	0·855
DBP (mm Hg)	69·66	2·25	72·30	2·88	72·81	2·35	0·586
Biochemical values (mmol/l)							
Total cholesterol	4·90	0·28	4·30	0·17	4·57	0·30	0·079
HDL-cholesterol	1·41	0·05	1·15	0·08	1·48	0·08	0·062
LDL-cholesterol	2·94	0·23	2·55	0·18	2·66	0·23	0·106
Triacylglycerol	1·21	0·15	1·31	0·29	0·95	0·15	0·769

Data are mean (sem). Differences in baseline characteristics between the three groups were determined using a general linear model. ^†^ Data on gender in three subjects were missing. DBP, diastolic blood pressure; SBP, systolic blood pressure.

### Consumption of fish oil supplements and oily fish altered the fatty acid profiles of circulating EV and RBC but had no effects on blood lipid profile

The proportions of EPA and DHA in both circulating EV and RBC were significantly increased following intervention with fish oil supplements and oily fish, with a substantial overall increase in total *n*-3 PUFA (Tables 3 and 4) The degree of incorporation of *n*-3 PUFA in the two groups was comparable, despite the fact that the fish oil supplements had a higher content of EPA + DHA (2 g *v*. 1 g) and a higher ratio of EPA to DHA (3:1 *v*. 1:1) than the oily fish meals (Table 1). There was a decrease in the proportion of total *n*-6 PUFA in RBC after the consumption of fish oil supplements or oily fish compared with the control group, but there was no difference between the fish oil supplements and control groups (Table 4). There were no accompanying changes in the proportions of any other fatty acids in either circulating EV or RBC following the consumption of fish oil supplements or oily fish (Tables 3 and 4). There was no significant effect of either oily fish or fish oil supplements on the blood lipid profile, although there were trends for a reduction in plasma triacylglycerol concentration and an increase in LDL-cholesterol concentration following intervention with both oily fish and fish oil supplements (online Supplementary Table 2).

**Table 3.** Effect of fish oil supplements and oily fish on the fatty acid composition of circulating EV (Mean values with their standard errors of the mean)

Fatty acids	Fish oil supplement		Oily fish		Control		*P* value (treatment)
	Before (wt%)	After (wt%)	Before (wt%)	After (wt%)	Before (wt%)	After (wt%)	
	Mean sem	Mean sem	Mean sem	Mean sem	Mean sem	Mean sem	
Palmitic acid (16:0)	24·83 ± 0·64	26·05 ± 0·61	25·86 ± 0·62	25·15 ± 0·55	24·50 ± 0·38	24·75 ± 0·52	0·372
Stearic acid (18:0)	9·66 ± 0·91	8·71 ± 0·55	8·93 ± 0·75	8·56 ± 0·57	9·83 ± 0·74	9·60 ± 0·80	0·570
Oleic acid (18:1, *n*-9)	30·28 ± 1·03	28·43 ± 0·66	30·34 ± 1·08	29·21 ± 1·07	30·52 ± 1·08	29·89 ± 0·93	0·805
Linoleic acid (18:2, *n*-6)	17·15 ± 0·44	17·48 ± 0·44	17·56 ± 0·77	18·58 ± 0·96	17·44 ± 1·05	17·97 ± 1·08	0·740
AA (20:4, *n*-6)	3·41 ± 0·21	2·90 ± 0·13	3·68 ± 0·20	3·05 ± 0·23	3·69 ± 0·16	3·44 ± 0·23	0·247
ALA (18:3, *n*-3)	0·25 ± 0·02	0·25 ± 0·01	0·25 ± 0·02	0·20 ± 0·02	0·23 ± 0·01	0·23 ± 0·02	0·386
EPA (20:5, *n*-3)	0·43 ± 0·03	1·07 ± 0·06	0·45 ± 0·03	1·03 ± 0·06	0·44 ± 0·03	0·48 ± 0·04	<0·001
DPA (22:5, *n*-3)	0·36 ± 0·02	0·39 ± 0·02	0·44 ± 0·02	0·42 ± 0·02	0·36 ± 0·02	0·31 ± 0·01	0·249
DHA (22:6, *n*-3)	1·06 ± 0·06	1·45 ± 0·07	1·10 ± 0·08	1·53 ± 0·06	0·91 ± 0·07	0·94 ± 0·10	**<**0·001
Total SFA	35·25 ± 1·06	35·54 ± 0·87	35·44 ± 1·03	34·32 ± 0·93	35·28 ± 0·70	35·37 ± 1·07	0·883
Total MUFA	33·34 ± 1·11	31·84 ± 0·76	33·72 ± 1·08	32·47 ± 1·03	33·93 ± 1·11	33·33 ± 0·95	0·735
Total *n*-3 PUFA	2·76 ± 0·13	4·11 ± 0·17	2·98 ± 0·13	4·17 ± 0·14	2·98 ± 0·19	2·88 ± 0·17	0·003
Total *n*-6 PUFA	22·79 ± 0·52	22·60 ± 0·50	23·54 ± 0·86	24·14 ± 1·04	23·59 ± 1·07	24·02 ± 1·20	0·465

Data are mean (sem). Differences in the fatty acid composition of circulating EV after intervention between the three groups were determined using a general linear model, including post hoc analysis with Bonferroni tests for treatment, period and treatment × time interaction with differences shown at *P* < 0·05. EPA: There was a significant effect of the treatment on the proportion of EPA in the circulating EV (*P* < 0·001) and significant time × treatment interaction (*P* < 0·001) with a significant effect of time (*P* < 0·001); the proportion of EPA has increased after fish oil and oily fish groups compared with the control group (both *P* < 0·001), but there was no difference between fish oil and oily fish groups. DHA: There was a significant effect of the treatment on the proportion of DHA in the circulating EV (*P* < 0·001) and significant time × treatment interaction (*P* = 0·004) with a significant effect of time (*P* < 0·001); the proportion of DHA has increased after fish oil and oily fish groups compared with the control group (*P* = 0·004 and *P* < 0·001, respectively), but there was no difference between fish oil and oily fish groups. Total *n*-3 PUFA: There was a significant effect of the treatment on the proportion of total *n*-3 PUFA in the circulating EV (*P* = 0·003) and significant time × treatment interaction (*P* < 0·001)with a significant effect of time (*P* < 0·001); the proportion of total *n*-3 PUFA has increased after fish oil and oily fish groups compared with the control group (*P* = 0·004 and *P* = 0·024, respectively), but there was no difference between fish oil and oily fish groups. AA, arachidonic acid; ALA, alpha-linolenic acid; DPA, docosapentaenoic acid; EV, extracellular vesicle.

**Table 4.** Effect of fish oil supplements and oily fish on the fatty acid composition of RBC (Mean values with their standard errors of the mean)

Fatty acids	Fish oil supplement		Oily fish		Control		*P* value (treatment)
	Before (wt%)	After (wt%)	Before (wt%)	After (wt%)	Before (wt%)	After (wt%)	
	Meansem	Meansem	Meansem	Meansem	Meansem	Meansem	
Palmitic acid (16:0)	24·42 ± 0·17	24·72 ± 0·24	24·78 ± 0·32	24·81 ± 0·32	24·39 ± 0·26	24·26 ± 0·19	0·403
Stearic acid (18:0)	12·59 ± 0·22	13·15 ± 0·21	12·51 ± 0·17	13·21 ± 0·29	12·93 ± 0·24	13·62 ± 0·32	0·265
Oleic acid (18:1, *n*-9)	18·60 ± 0·36	17·69 ± 0·29	18·97 ± 0·43	17·86 ± 0·40	18·70 ± 0·29	18·08 ± 0·27	0·812
Linoleic acid (18:2, *n*-6)	17·17 ± 0·40	15·00 ± 0·41	16·77 ± 0·79	14·66 ± 0·58	17·33 ± 0·50	15·88 ± 0·61	0·456
AA (20:4, *n*-6)	12·84 ± 0·41	12·48 ± 0·31	12·73 ± 0·41	12·97 ± 0·36	13·16 ± 0·37	14·13 ± 0·45	0·122
ALA (18:3, *n*-3)	0·45 ± 0·04	0·36 ± 0·02	0·46 ± 0·04	0·38 ± 0·02	0·39 ± 0·03	0·30 ± 0·03	0·174
EPA (20:5, *n*-3)	1·07 ± 0·07	2·77 ± 0·16	1·00 ± 0·07	2·22 ± 0·11	0·93 ± 0·08	1·20 ± 0·07	<0·001
DPA (22:5, *n*-3)	2·07 ± 0·06	2·98 ± 0·10	2·08 ± 0·09	2·63 ± 0·10	2·21 ± 0·12	2·44 ± 0·08	0·179
DHA (22:6, *n*-3)	3·92 ± 0·17	5·31 ± 0·16	3·72 ± 0·15	5·69 ± 0·19	3·51 ± 0·15	4·51 ± 0·18	0·005
Total SFA	37·15 ± 0·20	38·03 ± 0·23	37·45 ± 0·28	38·17 ± 0·26	37·47 ± 0·40	38·05 ± 0·32	0·781
Total MUFA	20·13 ± 0·41	18·66 ± 0·32	20·66 ± 0·50	18·83 ± 0·46	20·17 ± 0·37	19·12 ± 0·35	0·796
Total *n*-3 PUFA	7·74 ± 0·22	11·59 ± 0·33	7·46 ± 0·22	11·09 ± 0·33	7·20 ± 0·27	8·60 ± 0·26	<0·001
Total *n*-6 PUFA	32·36 ± 0·41	29·35 ± 0·47	31·71 ± 0·60	29·50 ± 0·57	32·48 ± 0·50	31·91 ± 0·48	0·037

Data are mean (sem). Differences in the fatty acid composition of RBC after intervention between the three groups were determined using a general linear model, including post hoc analysis with Bonferroni tests for treatment, period and treatment × time interaction with differences shown at *P* < 0·05. EPA: There was a significant effect of the treatment on the proportion of EPA in the circulating EV (*P* < 0·001) and significant time × treatment interaction (*P* < 0·001) with a significant effect of time (*P* < 0·001); the proportion of EPA has increased after fish oil and oily fish groups compared with the control group (both *P* < 0·001), but there was no difference between fish oil and oily fish groups. DHA: There was a significant effect of the treatment on the proportion of EPA in the circulating EV (*P* = 0·005) and significant time × treatment interaction (*P* = 0·001) with a significant effect of time (*P* < 0·001); the proportion of EPA has increased after fish oil and oily fish groups compared with the control group (*P* = 0·022 and *P* = 0·007, respectively), but there was no difference between fish oil and oily fish groups. Total *n*-3 PUFA: There was a significant effect of the treatment on the proportion of total *n*-3 PUFA in the circulating EV (*P* < 0·001) and significant time × treatment interaction (*P* < 0·001)with a significant effect of time (*P* < 0·001); the proportion of total *n*-3 PUFA has increased after fish oil and oily fish groups compared with the control group (*P* < 0·001 and *P* = 0·001, respectively), but there was no difference between fish oil and oily fish groups. Total *n*-6 PUFA: There was a significant effect of the treatment on the proportion of total *n*-6 PUFA in the circulating EV (*P* = 0·037) and significant time × treatment interaction (*P* = 0·008)with a significant effect of time (*P* < 0·001); the proportion of total *n*-6 PUFA has increased after oily fish group compared with the control group (*P* = 0·043), but there was no difference between fish oil control groups. AA, arachidonic acid; ALA, alpha-linolenic acid; DPA, docosapentaenoic acid; EV, extracellular vesicle.

### Fish oil supplements, but not oily fish, decreased numbers of circulating EV

**Figure 1.**
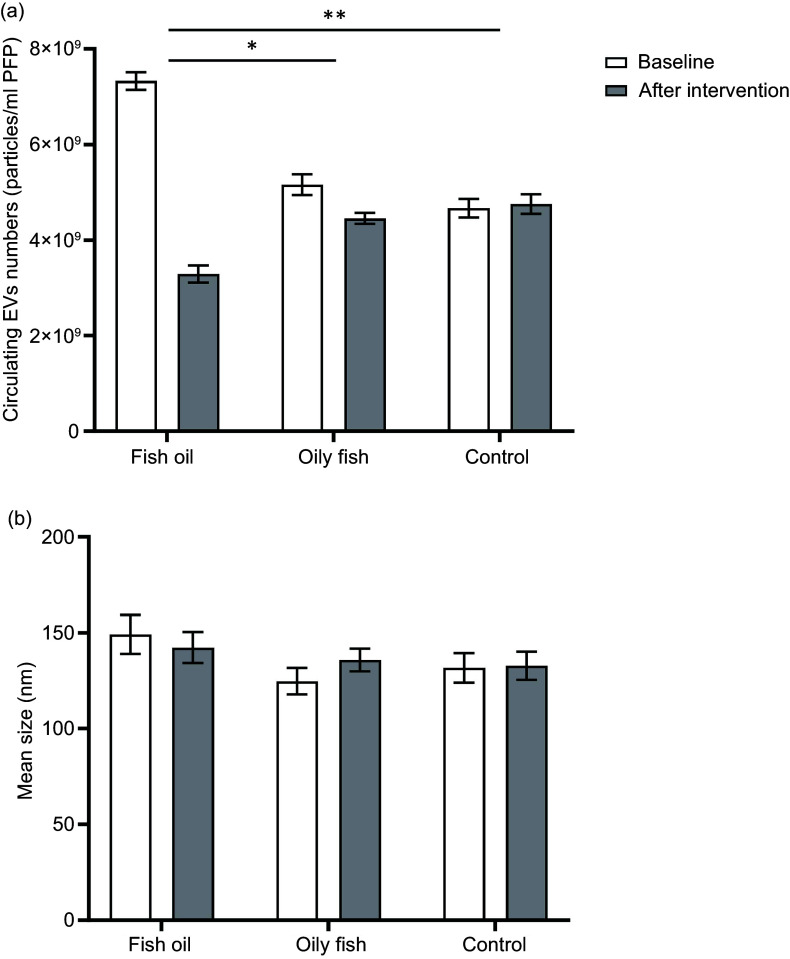
Effects of oily fish and fish oil supplements on the numbers and mean size of circulating EV determined by NTA. Data were analysed by using a general linear model, including post hoc analysis with Bonferroni tests for treatment, period and treatment × time interaction with differences shown at *P* < 0·05. (a) There was a significant effect of treatment on EV number (*P* = 0·004) and significant time × treatment interaction (*P* < 0·001), with a significant effect of time (*P* < 0·001); EV numbers were significantly decreased after fish oil supplement compared with oily fish (*P* = 0·023) and control (*P* = 0·007). (b) There were no statistically significant effects of treatment and time interaction on EV size (*P* = 0·299 and *P* = 0·389, respectively). *P < 0·05 and **P < 0·01. EV, extracellular vesicles; PFP, platelet-free plasma.

Supplementation with fish oil significantly decreased the numbers of circulating EV, whereas the consumption of oily fish had no effect (Figure 1(a)). There was no effect of either oily fish or fish oil supplements on the mean size of the EV population (Figure 1(b)) or on the numbers of PS-positive circulating EV, PDEV and endothelial-derived EV (online Supplementary Table 2).

### Fish oil supplements, but not oily fish, reduce the ability of EV to support thrombin generation

**Figure 2.**
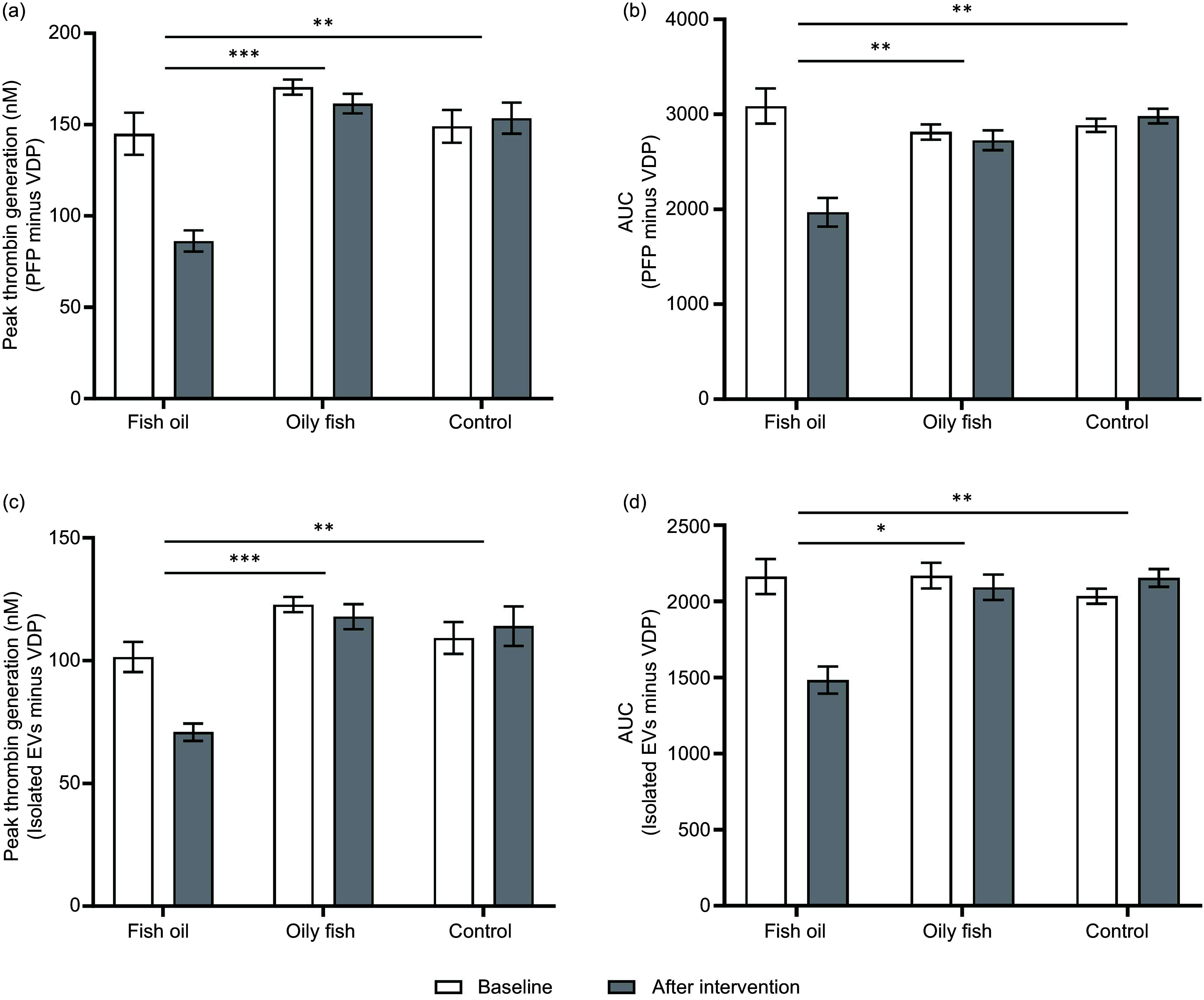
Effects of oily fish and fish oil supplements on circulating EV-supported thrombin generation. Data were analysed by using a general linear model, including post hoc analysis with Bonferroni tests for treatment, period and treatment × time interaction with differences shown at *P* < 0·05. (a) There was a significant effect of treatment on EV-supported (PFP minus VDP) thrombin peak concentration (*P* < 0·001) and significant time × treatment interaction (*P* = 0·001), with a significant effect of time (*P* = 0·010), in which fish oil supplement significantly decreased thrombin peak concentration compared with oily fish (*P* < 0·001) and control (*P* = 0·001). (b) There was a significant effect of the treatment on EV-supported (PFP minus VDP) thrombin AUC (*P* < 0·001) and significant time × treatment interaction (*P* < 0·001), with a significant effect of time (*P* = 0·007), in which fish oil supplement significantly decreased thrombin AUC compared with oily fish (*P* = 0·001) and control (*P* = 0·004). (c) There was a significant effect of the treatment on EV-supported (isolated EV minus VDP) thrombin peak concentration (*P* < 0·001) and significant time × treatment interaction (*P* < 0·001), with a significant effect of time (*P* < 0·001), in which fish oil supplement significantly decreased thrombin peak concentration compared with oily fish (*P* < 0·001) and control (*P* = 0·001). (d) There was a significant effect of the treatment on EV-supported (Isolated EVs minus VDP) thrombin AUC (*P* = 0·001) and significant time × treatment interaction (*P* < 0·001), with a significant effect of time (*P* < 0·001), in which fish oil supplement significantly decreased thrombin AUC compared with oily fish (*P* = 0·033) and control (*P* = 0·001). *P < 0·05, **P < 0·01, ***P < 0·001. EV, extracellular vesicles; PFP, platelet-free plasma; VDP, vesicles-depleted plasma.

The thrombogenicity of EV following intervention was analysed using two approaches. In the first approach, the difference in thrombin generation between PFP from study subjects and pooled VFP from healthy subjects indicated the degree to which EV influenced tissue factor (TF)-stimulated thrombin generation in plasma before and after the intervention. PFP from subjects supplemented with fish oil had significantly lower thrombogenic capacity than that from subjects in the oily fish or control groups, as determined by peak thrombin concentration, AUC (Figure 2(a) and (b)) and velocity index (treatment: *P* = 0·015; time: *P* = 0·276; time × treatment interaction: *P* = 0·016). The second approach, where SEC-isolated EV from study subjects were added to pooled VFP from healthy subjects, demonstrated that EV from subjects supplemented with fish oil had significantly lower thrombogenic capacity than those from subjects in the oily fish or control groups, as determined by peak thrombin concentration and AUC (Figure 2(c) and (d)).

### Fish oil supplements and oily fish have no effect on the ability of extracellular vesicle to induce clot formation and lysis

There was no significant effect of either oily fish or fish oil supplements on clot formation or lysis supported by isolated EV from subjects, as determined by AUC and time to full lysis (online Supplementary Table 2).

### Fatty acid profiles of circulating extracellular vesicle and RBC are associated with numbers and coagulatory activity of circulating extracellular vesicle

**Figure 3.**
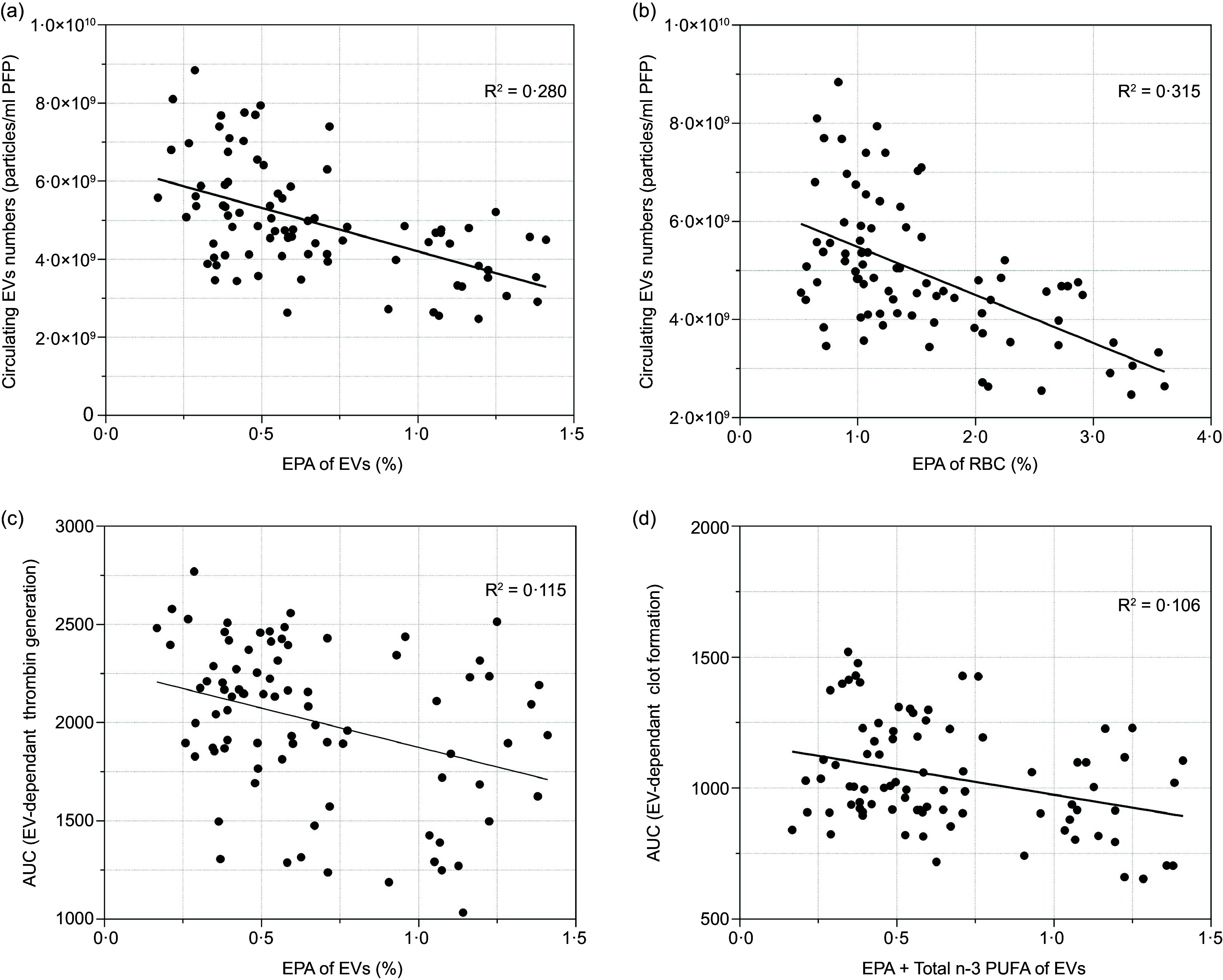
Association between fatty acid profiles of circulating EV and RBC with numbers and coagulatory activity of circulating EV. Data were analysed by using a stepwise multivariate regression model to identify independent predictors of EV parameters. (a) The proportion of EPA in circulating EV independently explained 28·0 % of the variance for circulating EV numbers. (b) The proportion of EPA in RBC independently explained 31·5 % of the variance for circulating EV numbers. (c) The proportion of EPA in circulating EV independently explained 11·5 % of the variance for AUC in EV-dependent thrombin generation. (d) The proportion of EPA in circulating EV independently explained 10·6 % of the variance for AUC in EV-dependent clot formation. EV, extracellular vesicles.

The proportions of EPA, DHA, ETA and total *n*-3 PUFA in circulating EV were significantly associated with the numbers of circulating EV (online Supplementary Table 3), and the proportions of EPA, DHA, DPA, stearic acid, linoleic acid, total *n*-3 PUFA, total MUFA and total *n*-6 PUFA in RBC were significantly correlated with circulating EV numbers (online Supplementary Table 4). Stepwise regression analysis suggested that the proportions of EPA in circulating EV and RBC explained 28·0 % and 31·5 % of the variance for circulating EV numbers, respectively (Figures 3(a) and (b)).

The proportions of EPA, ALA, stearic acid, linoleic acid and total *n*-3 PUFA in circulating EV were significantly associated with EV-supported thrombin generation parameters. EPA and total *n*-3 PUFA in circulating EV were also significantly associated with EV-supported clot formation parameters (online Supplementary Table 3). The proportion of EPA in circulating EV independently predicted 11·5 % of the variance for EV-supported thrombin generation (AUC) and 10·6 % of the variance in EV-supported clot formation (AUC), respectively (Figures 3(c) and (d)). Furthermore, the proportions of EPA, DHA, DPA, oleic acid, total *n*-3 PUFA, total MUFA and total *n*-6 PUFA in RBC were associated with EV-supported thrombin generation and clot formation parameters (online Supplementary Table 4). The proportion of EPA in RBC explained 14·3 % of the variance in EV-supported thrombin generation (AUC) and 13·6 % of the variance in EV-supported clot formation (AUC), respectively (data not shown).

## Discussion

This study demonstrated that fish oil supplements are more effective than oily fish in decreasing numbers of circulating EV and reducing their procoagulant activity, and that these effects appear to be attributable to the additional 1 g/d EPA delivered in the fish oil supplements. Consumption of both oily fish and fish oil supplements increased the proportions of EPA and DHA in circulating EV and RBC to a similar degree, despite the dose of *n*-3 PUFA in the oily fish meals being half that provided by the supplements. The proportion of EPA in circulating EV and RBC was strongly associated with circulating EV numbers and EV-stimulated thrombin generation, which also suggests that EPA, rather than DHA, was driving the effects.

The dramatic reduction in the numbers of circulating EV after fish oil supplements confirms some previous observations^(16–20)^ and the reduction in thrombin generation supported by EV is consistent with a small number of studies in healthy individuals^(21,29)^, or post-myocardial infarction patients^(19)^. However, not all studies agree; healthy subjects consuming 1·2 g/d EPA + DHA for 4 weeks did not experience an alteration in the number of circulating PS-positive EV^(30)^, and PDEV numbers were not significantly altered after an 8-week intervention with 1·5 g EPA + DHA per day^(20)^. Furthermore, to our knowledge, this is the first study comparing the effects of *n*-3 PUFA in the form of oily fish, at a level achievable in the diet, with fish oil supplements on EV numbers and functional activity. Ideally, the two treatments would have delivered equivalent doses and ratios of EPA and DHA, but the design of the study was dependent on the limited options and availability of oily fish meals and supplements. As a result, the doses of EPA and DHA delivered in the supplements were approximately 1·5 g/d and 0·5 g/d respectively, compared with approximately 0·6 g/d and 0·4 g/d, respectively, in the oily fish meals.

A small number of studies have compared the intervention with *n*-3 PUFA in the form of oily fish and fish oil capsules on the incorporation of EPA and DHA into plasma and cellular lipids. Harris *et al.* demonstrated the equivalent incorporation of EPA and DHA into RBC and plasma phospholipids following consumption of oily fish or fish oil capsules providing equal amounts of EPA and DHA^(31)^. Another study demonstrated that EPA content was higher in RBC and platelets following supplementation, but DHA content was higher in RBC and platelets following intervention with salmon, and this was most likely due to a ratio of EPA:DHA of 1·6:1 in the supplement group compared with 1:2·4 in the salmon group, explaining the greater accumulation of DHA in the salmon group^(32)^. The current study reports a similar degree of incorporation of EPA, DHA and total *n*-3 PUFA into both RBC and EV in the fish oil and oily fish groups, even though the supplements provided a higher EPA: DHA ratio compared with oily fish meals. It should also be noted that *n*-3 PUFA are present in the ethyl ester form in supplements, whereas they are present as triglycerides and phospholipids in oily fish, where they are considered to have superior bioavailability, so comparisons between the two should take this into account^(33,34)^.

The fact that fish oil supplements, but not oily fish, decreased EV numbers and their thrombogenic capacity in this study suggests that the additional 1 g/d EPA delivered in the fish oil supplements against an equivalent DHA background is required for the effects. There is limited insight into the differential effects of EPA and DHA on EV properties, but one study reported that thrombin generation stimulated by PDEV was decreased after a 24-h intervention with an EPA-rich oil (1 g EPA with an EPA/DHA ratio of 5:1), but not a DHA-rich oil (1 g DHA with an EPA/DHA ratio of 1:5) or a placebo treatment^(29)^. The current study demonstrated that the proportion of EPA in circulating EV and RBC independently predicted circulating EV numbers and EV-supported thrombin generation, supporting the argument that EPA is the main driver of the effects of *n*-3 PUFA on EV function. Nomura *et al.* conducted three trials, which provided patients with hyperlipidaemia and type 2 diabetes with pure EPA at a dose of 1·8 g/d for 4 weeks^(16)^ or 6 months^(17)^, or combined with 2 mg/d pitavastatin for 6 months^(18)^. They demonstrated significant decreases in numbers of PDEV, monocyte-derived EV^(16,18)^ and endothelial-derived EV^(17)^ after EPA supplementation.

The exact mechanisms by which *n*-3 PUFA decrease numbers of circulating EV and inhibit their coagulatory function are not fully understood, but it is well appreciated that the incorporation of *n*-3 PUFA into cell membrane phospholipids may play a fundamental role in modulating the lipid composition and function of cells, thereby altering the generation and behaviour of circulating EV^(1,2,35–37)^. Externalisation of PS in the outer leaflet during cell activation is a key step of EV generation and a key contributor to the thrombin-generating capacity of EV, and it has been demonstrated to be inhibited by flaxseed oil-derived *n*-3 PUFA^(38)^. Larson and colleagues also reported that PS exposure by platelets was reduced by approximately 50 % after 28 d of *n*-3 PUFA supplementation in healthy subjects^(39)^. Underlying mechanisms may also involve lipid rafts, which are involved in EV formation, cargo loading and fusion with target cells, and have been reported to be altered by *n*-3 PUFA, giving rise to the suggestion that this alteration may disrupt EV shedding and behaviour^(40,41)^. The decrease in total *n*-6 PUFA in RBC reported in this study may provide an additional explanation. *n*-3 and *n*-6 PUFA are metabolised in a competitive manner, and *n*-6 PUFA-derived lipid mediators enhance platelet aggregation, thrombin generation and inflammation. Thus, the incorporation of *n*-3 PUFA into cell membranes may decrease EV generation and coagulatory function through modification of the lipid mediator profile^(42,43)^.

The favourable effects of EPA on EV have previously been attributed to more rapid incorporation of EPA into the phospholipids of cell membranes compared with DHA^(35–37,44)^. For example, the consumption of 1 g/d EPA for 3 d significantly increased concentrations of EPA in plasma and RBC phospholipids, while a similar increase in DHA required 6 d of consumption at a dose of 1 g/d^(36)^. Similarly, enrichment of EPA in plasma phospholipids occurred earlier and was more marked and more dose-dependent than that of DHA when supplemented at doses ranging from 1–4 g/d^(37,44)^. Although there was a trend towards a higher EPA content in RBC in the fish oil group compared with the oily fish group in this study, this was not statistically significant and it may be that the unique effects of EPA are not limited to its incorporation into plasma membranes and that similar incorporation in the fish oil and oily fish groups does not therefore imply that the effects will be the same. They could, for example, be related to the effects of EPA on cardiometabolic factors, such as platelet activation and endothelial dysfunction, and these in turn affect EV generation and function^(45–50)^. The behaviour of EV is dependent on its cargo, which may also be modified by fatty acid supplementation, and this may influence not only procoagulatory activity, but also fibrinolysis^(51)^.

The strengths of this study include the fact that it explores the effects of consumption of oily fish at a level achievable in the diet on EV numbers and thrombogenic capacity and suggests that a higher dose of EPA than is possible through dietary intervention may be required to achieve significant effects. The main limitations of the study relate to it being a parallel trial, which potentially introduces confounding variables, such as the influence of biological sex on outcomes^(52)^, and differences in baseline circulating EV numbers between the fish oil and control groups, which could influence the interpretation of the findings. Future studies may benefit from a cross-over design and further exploration of sex-specific effects to validate and expand upon these conclusions. It is also notable that there were no effects of *n*-3 PUFA on EV subtypes, despite the dramatic effects on total EV numbers. EV subtypes were analysed by flow cytometry, which has a detection limit of 200 nm, which means that they only represent EV subtypes larger than 200 nm, whereas total EV were analysed by nanoparticle tracking analysis. Thus, flow cytometry, and therefore subtype analysis, only represents a small proportion of total EV. The interpretation is that fish oil affected smaller EV, but it is not possible to identify which subtypes are altered using currently available techniques.

In conclusion, this study demonstrates that fish oil supplements delivering a high dose of EPA were more effective than oily fish in altering the number and thrombogenicity of EV. EPA therefore appears to be a key factor driving alterations in EV number and function, an observation supported by a strong association between the EPA content of EV and their biological activity. The study suggests that increasing EPA intake beyond the current dietary guidelines for oily fish consumption may offer additional benefits, particularly in relation to EV numbers and functions.

## Supporting information

Sharman et al. supplementary material 1 Sharman et al. supplementary material

Sharman et al. supplementary material 2 Sharman et al. supplementary material

## Supplementary material

For supplementary material/s referred to in this article, please visit https://doi.org/10.1017/S0007114525000625
