# Supplementary material for: High-dose fish oil supplements are more effective than oily fish in altering the number and function of extracellular vesicles in healthy human subjects: a randomised, double-blind, placebo-controlled, parallel trial
Source: Br J Nutr. 2025 Mar 21;133(7):934–44. doi: 10.1017/S0007114525000625 (PMC12198344; doi:10.1017/S0007114525000625)
Supplement: Sharman et al. supplementary material 1 — Sharman et al. supplementary material [file S0007114525000625sup001.docx]

**High dose fish oil supplements are more effective than oily fish in altering the number and function of extracellular vesicles in healthy human subjects: A randomized, double-blind, placebo-controlled, parallel trial**

Sharman A. *et al.*

**Online Supplementary Material**

# **Supplementary Figures**

**Supplementary Figure 1.** Participant flow

Assessed for eligibility (n = 100)

Excluded (n =42)

♦ Not meeting inclusion criteria (n = 40)

♦ Unable to participate (n = 2)

Enrolment

Randomized (n = 58)

Allocate to Control group (n = 19)

Allocate to Fish oil group (n = 20)

Allocate to Oily fish group (n = 19)

Allocation & study conduct

♦ Received allocated intervention (n=13)

♦Lost to follow up (n = 3)

♦ Post sample not collected (n = 3)

Lost to follow up (n = 2)

♦ Post sample not collected (n = 3)

♦ Received allocated intervention (n=15)

♦Lost to follow up (n = 2)

♦ Post sample not collected (n = 3)

Lost to follow up (n = 2)

♦ Post sample not collected (n = 3)

♦ Received allocated intervention (n=14)

♦Lost to follow up (n = 2)

♦ Post sample not collected (n = 3)

Analyzed (n = 13)

Analyzed (n = 15)

Analyzed (n = 14)

Analysis

# **Supplementary Tables**

**Supplementary Table 1.** Scoring tool for recruitment

| **CVD risk factors** | **1 point** | | **2 points** | |
| --- | --- | --- | --- | --- |
|  | **Males** | **Females** | **Males** | **Females** |
| Total Cholesterol (mmol/L) | 5.18-6.21 | 5.18-6.21 | 6.22-7.99 | 6.22-7.99 |
| HDL Cholesterol (mmol/L) | 0.91-1.16 | 1.17-1.29 | <90 | <1.16 |
| Glucose (mmol/L) | 6.00-6.99 | 6.00-6.99 | NA | NA |
| BMI (kg/m^2^) | 25.5-29.9 | 25.5-29.9 | 30.0-39.9 | 30.0-39.9 |
| Waist circumference (cm) | >94 | >80 | >102 | >88 |
| SBP (mm Hg) | 130-139 | 130-139 | 140-159 | 140-159 |
| DBP (mm Hg) | NA | NA | 90-99 | 90-99 |
| First degree relative diagnosed with MI or T2D (age of diagnosis) (y) | NA | NA | <55 y in male relatives;  <65 y in female relatives | <55 y in male relatives;  <65 y in female relatives |

The scoring tool was based on the Framingham Risk Score system (22), adapted by Chong *et al.* (23) to include a score for family history of MI or T2D. Participants were required to score ≥ 2 points to have an RR ≥ 1.5 of developing CVD, which could be achieved through a combination of CVD risk factors. *MI, myocardial infraction; T2D, type 2 diabetes; NA, not applicable.*

**Supplementary Table 2.** Effect of fish oil supplements and oily fish on the plasma lipid profile, numbers of EV subtypes, and clot formation and lysis supported by circulating EVs

|  | **Fish oil supplement** | | **Oily fish** | | | | **Control** | | **P value**  **(treatment)** |
| --- | --- | --- | --- | --- | --- | --- | --- | --- | --- |
|  | Before (wt%) | After (wt%) | | Before (wt%) | After (wt%) | Before (wt%) | | After (wt%) |  |
|  |  |  | |  |  |  | |  |  |
| **Plasma lipid profile(mmol/L)** | |  | |  |  |  | |  |  |
| Total cholesterol | 4.90±0.28 | 4.99±0.25 | | 4.30±0.17 | 4.70±0.17 | 4.57±0.30 | | 4.79±0.35 | 0.218 |
| LDL-C cholesterol | 2.94±0.23 | 3.11±0.21 | | 2.55±0.18 | 2.99±0.15 | 2.66±0.23 | | 2.84±0.30 | 0.218 |
| HDL-C cholesterol | 1.41±0.05 | 1.43±0.10 | | 1.15±0.08 | 1.30±0.08 | 1.48±0.08 | | 1.54±0.06 | 0.173 |
| Triacylglycerol | 1.21±0.15 | 0.99±0.07 | | 1.31±0.29 | 0.89±0.15 | 0.95±0.15 | | 0.90±0.19 | 0.988 |
|  | |  | |  |  |  | |  |  |
| **EV subtype numbers (particles/ ml PFP)** | |  | |  |  |  | |  |  |
| PS-positive EVs | 1.2E+8 ±1.6E+7 | 6.2E+07±9.3E+6 | | 1.2E+8±1.4E+7 | 6.9E+7±1.3E+7 | 1.2E+8±4.1E+7 | | 1.2E+8±4.1E+7 | 0.976 |
| PDEVs | 5.6E+7±9.8E+6 | 3.39E+7±9.9E+6 | | 7.9E+7±1.3E+7 | 4.0E+7± 7.1E+6 | 8.6E+7±4.0E+7 | | 4.2E+7± 7.4E+6 | 0.567 |
| EDEVs | 1.8E+7±2.1E+6 | 1.2E+7±1.5E+6 | | 1.4E+7±4.3E+6 | 8.1E+6 ±1.9E+6 | 1.4E+7±4.5E+6 | | 8.3E+6±1.2E+6 | 0.387 |
| **Clot formation and lysis** | |  | |  |  |  | |  |  |
| Time to full lysis (min) | 468.93±121.91 | 503.88±128.75 | | 448.54±112.71 | 477.66±116.51 | 502.42±99.01 | | 533.00±121.64 | 0.192 |
|  |  |  | |  |  |  | |  |  |
| AUC | 1073.71±163.68 | 825.71±133.99 | | 1232.35±183.34 | 1102.91±179.49 | 1023.21±199.71 | | 999.37±146.19 | 0.075 |

Data are mean ± SEM. Differences in the plasma lipid profile, numbers of EV subtypes, and clot formation and lysis supported by circulating EVs between the three groups were determined using a general linear model, including post-hoc analysis with Bonferroni tests for treatment, period and treatment*time interaction with differences shown at *P* < 0.05. There was no effect of either oily fish or fish oil supplements on the numbers of EV subtypes and plasma lipid profile. *AUC, area under curve;* *EVs, extracellular vesicles; EDEVs, endothelial-derived extracellular vesicles; HDL-C, high-density lipoprotein cholesterol; LDL-C, low-density lipoprotein cholesterol; PDEVs, platelet-derived extracellular vesicles; PS-positive EVs, phosphatidylserine positive extracellular vesicles.*

**Supplementary Table 3.** Associations between fatty acid profiles of circulating EVs with numbers and coagulatory activity of circulating EVs

Pearson’s correlation coefficient or Spearman's correlation coefficient was conducted to examine the associations between fatty acid profiles of circulating EVs with numbers and coagulatory activity of circulating EVs**.** *. Correlation is significant at the 0.05 level (2-tailed). **. Correlation is significant at the 0.01 level (2-tailed). *AA, arachidonic acid;* *ALA, alpha-linolenic acid;* *AUC, area under curve; EPA, eicosapentaenoic acid; EVs, extracellular vesicles; DPA, docosapentaenoic acid; DHA, docosahexaenoic acid; MUFAs, monounsaturated fatty acids;* *PUFAs, polyunsaturated fatty acids; SFAs, saturated fatty acids.*

| **Fatty acids** |  | **EV numbers** | **EV-dependent thrombin generation**  **(Isolated EVs minus VDP)** | | | | **EV-dependent clot formation** | |
| --- | --- | --- | --- | --- | --- | --- | --- | --- |
|  |  |  | Lag time | Peak thrombin concentration | Velocity index | AUC | Time to full lysis | AUC |
| Palmitic acid (16:0) | *r* | -.020 | .110 | -.033 | -.119 | -.007 | .131 | .057 |
|  | *p* | .854 | .321 | .764 | .281 | .947 | .236 | .606 |
| Stearic acid (18:0) | *r* | -.064 | **.290**** | -.003 | -.117 | .090 | .064 | .040 |
|  | *p* | .561 | **.008** | .981 | .289 | .418 | .562 | .721 |
| Oleic acid (18:1, n-9) | *r* | .201 | -.055 | .126 | .186 | .102 | -.119 | .110 |
|  | *p* | .067 | .618 | .255 | .091 | .354 | .280 | .317 |
| Linoleic acid (18:2, n-6) | *r* | -.080 | **-.218*** | .040 | -.062 | -.054 | -.111 | -.056 |
|  | *p* | .469 | **.047** | .721 | .577 | .623 | .314 | .616 |
| AA (20:4, n-6) | *r* | .124 | .164 | .114 | .270* | .116 | -.112 | -.092 |
|  | *p* | .261 | .137 | .303 | .013 | .294 | .312 | .403 |
| ALA (18:3, n-3) | *r* | .020 | **.233*** | -.156 | -.122 | -.185 | .003 | .154 |
|  | *p* | .857 | **.033** | .156 | .269 | .091 | .981 | .161 |
| EPA (20:5, n-3) | *r* | **-.525**** | **-.114** | **-.286**** | **-.080** | **-.316**** | -.013 | **-.275*** |
|  | *p* | **<.001** | **.300** | **.008** | **.469** | **.003** | .909 | **.011** |
| DPA (22:5, n-3) | *r* | .017 | .149 | .081 | .149 | .121 | -.184 | .164 |
|  | *p* | .876 | .175 | .463 | .176 | .273 | .094 | .137 |
| DHA (22:6, n-3) | *r* | **-.244*** | -.073 | -.079 | .037 | -.210 | -.003 | -.129 |
|  | *p* | **.025** | .510 | .474 | .739 | .055 | .981 | .242 |
| Total SFA | *r* | -.028 | **.341**** | .033 | **-.220*** | .122 | .150 | .083 |
|  | *p* | .800 | **.001** | .764 | **.045** | .267 | .172 | .452 |
| Total MUFA | *r* | .177 | -.043 | .128 | .209 | .098 | -.076 | .091 |
|  | *p* | .108 | .696 | .246 | .057 | .375 | .490 | .411 |
| Total n-3 PUFA | *r* | **-.492**** | -.036 | -.153 | -.076 | **-.317**** | -.045 | -.085 |
|  | *p* | **<.001** | .744 | .165 | .494 | **.003** | .685 | .444 |
| Total n-6 PUFA | *r* | -.055 | -.194 | .066 | .028 | .001 | -.097 | -.092 |
|  | *p* | .617 | .078 | .550 | .803 | .991 | .378 | .403 |

**Supplementary Table 4.** Associations between fatty acid profiles of RBCs with numbers and coagulatory activity of circulating EVs

Pearson’s correlation coefficient or Spearman's correlation coefficient was conducted to examine the associations between fatty acid profiles of RBCs with numbers and coagulatory activity of circulating EVs**.** *. Correlation is significant at the 0.05 level (2-tailed). **. Correlation is significant at the 0.01 level (2-tailed). *AA, arachidonic acid; ALA, alpha-linolenic acid; AUC, area under curve; EPA, eicosapentaenoic acid; EVs, extracellular vesicles; DPA, docosapentaenoic acid; DHA, docosahexaenoic acid; MUFAs, monounsaturated fatty acids; PUFAs, polyunsaturated fatty acids; SFAs, saturated fatty acids.*

| **Fatty acids** |  | **EV numbers** | **EV-dependent thrombin generation**  **(Isolated EVs minus VDP)** | | | | **EV-dependent clot formation** | |
| --- | --- | --- | --- | --- | --- | --- | --- | --- |
|  |  |  | Lag time | Peak thrombin concentration | Velocity index | AUC | Time to full lysis | AUC |
| Palmitic acid (16:0) | *r* | -.014 | .124 | -.026 | -.004 | -.088 | .001 | -.046 |
|  | *p* | .899 | .268 | .814 | .975 | .431 | .990 | .683 |
| Stearic acid (18:0) | *r* | **-.221*** | .097 | .066 | -.005 | -.078 | .161 | -.197 |
|  | *p* | **.046** | .388 | .554 | .967 | .487 | .149 | .077 |
| Oleic acid (18:1, n-9) | *r* | .211 | .021 | -.003 | .107 | .072 | -.045 | **.271*** |
|  | *p* | .058 | .852 | .977 | .339 | .520 | .685 | **.014** |
| Linoleic acid (18:2, n-6) | *r* | **.270*** | -.011 | .095 | -.101 | .161 | -.157 | .146 |
|  | *p* | **.014** | .918 | .396 | .365 | .149 | .158 | .190 |
| AA (20:4, n-6) | *r* | .065 | .063 | .119 | .194 | .077 | .124 | -.041 |
|  | *p* | .560 | .573 | .286 | .080 | .493 | .267 | .714 |
| ALA (18:3, n-3) | *r* | .164 | -.058 | -.023 | .062 | .017 | -.016 | .167 |
|  | *p* | .142 | .603 | .838 | .578 | .878 | .885 | .134 |
| EPA (20:5, n-3) | *r* | **-.511**** | -.119 | **-.301**** | -.128 | **-.255*** | .147 | **-.303**** |
|  | *p* | **<.001** | .288 | **.006** | .250 | **.021** | .189 | **.006** |
| DPA (22:5, n-3) | *r* | **-.444*** | -.067 | **-.285**** | -.132 | **-.278*** | .126 | **-.332**** |
|  | *p* | **<.001** | .550 | **.009** | .237 | **.011** | .261 | **.002** |
| DHA (22:6, n-3) | *r* | **-.435**** | -.172 | -.144 | -.157 | **-.222*** | .037 | -.187 |
|  | *p* | **<.001** | .123 | .197 | .158 | **.045** | .741 | .092 |
| Total SFA | *r* | -.213 | .185 | .001 | .097 | -.154 | .132 | -.139 |
|  | *p* | .055 | .096 | .920 | .385 | .166 | .237 | .213 |
| Total MUFA | *r* | .269* | .065 | .022 | .107 | .088 | -.038 | **.299**** |
|  | *p* | .015 | .559 | .846 | .340 | .431 | .733 | **.006** |
| Total n-3 PUFA | *r* | **-.506**** | -.164 | **-.235*** | -.151 | **-.279*** | .151 | **-.261*** |
|  | *p* | **<.001** | .141 | **.003** | .175 | **.011** | .174 | **.018** |
| Total n-6 PUFA | *r* | **.300**** | .004 | .189 | .041 | **.253*** | -.094 | .060 |
|  | *p* | **.006** | .969 | .088 | .714 | **.022** | .402 | .593 |
