# Supplementary figures and images for: High-dose fish oil supplements are more effective than oily fish in altering the number and function of extracellular vesicles in healthy human subjects: a randomised, double-blind, placebo-controlled, parallel trial
Source: Br J Nutr. 2025 Mar 21;133(7):934–44. doi: 10.1017/S0007114525000625 (PMC12198344; doi:10.1017/S0007114525000625)

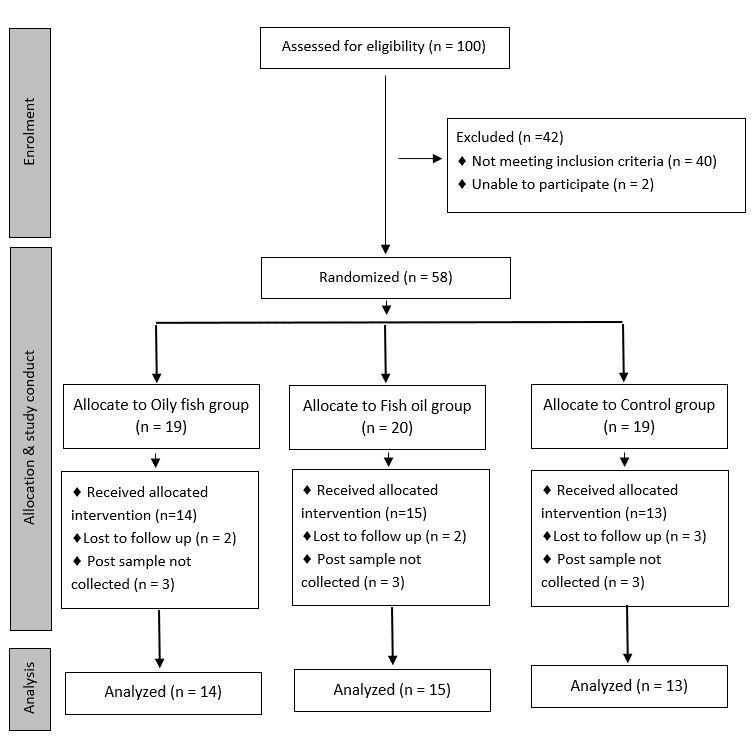

Supplement: Sharman et al. supplementary material 2 — Sharman et al. supplementary material [file S0007114525000625sup002.zip › Supplementary Figure 1.png]
